# Post-chikungunya chronic arthralgia in Burkina Faso: Frequency and associated factors

**DOI:** 10.1371/journal.pntd.0014251

**Published:** 2026-04-24

**Authors:** Fulgence Kaboré, Wendpanga Jean Emmanuel Savadogo, Yannick Laurent Tchenadoyo Bayala, Camille Sompougdou, Charles Sougué, Aboubakar Ouedraogo, Yamyellé Enselme Zongo, Wendlassida Joelle Stéphanie Zabsonré/ Tiendrébeogo, Dieu-Donné Ouédraogo

**Affiliations:** 1 Rheumatology Department, Bogodogo University Hospital Center, Ouagadougou, Burkina Faso; 2 Rheumatology Department, Regional University Hospital Center of Ouahigouya, Ouahigouya, Burkina Faso; 3 Internal Medicine Department, Sourou Sanou University Hospital Center, Bobo-Dioulasso, Burkina Faso; Colorado State University, UNITED STATES OF AMERICA

## Abstract

**Introduction:**

Chikungunya is an arboviral disease characterized by acute osteoarticular manifestations with a risk of progression to chronicity. African data on post-chikungunya chronic arthralgia are scarce. This study aimed to identify factors associated with post-chikungunya chronic arthralgia in Burkina Faso.

**Patients and methods:**

This was a descriptive and analytical cross-sectional study with ambispective data collection, conducted from April 22 to December 28, 2024, at the Medical Center of Pouytenga. All patients aged 16 years and older with RT-PCR- confirmed chikungunya virus infection were included. The evaluation comprised an initial clinical phase and telephone follow-up at 6 months of evolution.

**Results:**

We enrolled 174 patients, of whom 111 met the inclusion criteria. The mean age was 31.18 years ± 11.27 years with a female predominance of 66.67% (n = 74). Initial osteoarticular manifestations affected 92.79% of patients (n = 103), predominantly inflammatory arthralgia in 92.23% (n = 95) and symmetric polyarticular involvement in 73.79% (n = 76). At 6 months of evolution, 28.16% (n = 29) of patients had post-chikungunya chronic arthralgia, with persistence in the ankles in 65.52% of cases (n = 19). Multivariate analysis identified two significantly associated risk factors for chronic arthralgia were age greater than 31 years (OR = 6.22; 95% CI [2.35–16.47]; p < 0.001) and dengue coinfection (OR = 18.67; 95% CI [1.74–199.70]; p = 0.016]; p = 0.024).

**Conclusion:**

Our study found that 28.16% of patients develop post-chikungunya chronic arthralgia at 6 months post infection. Age above 31 years was demonstrated to be a significant predictor of disease chronicity, while the potential role of dengue co-infection, observed in only 4 patients with wide confidence intervals, requires further investigation.

## Author summary

Chikungunya is a mosquito-borne viral disease that causes sudden fever and severe joint pain. While most patients recover within weeks, some continue to experience joint pain for months or even years. Data on this chronic phase are very limited in Africa. We conducted a study in Burkina Faso to determine how often long-lasting joint pain occurs after chikungunya infection and what factors increase the risk.

Among 111 patients with laboratory-confirmed chikungunya, almost all had joint symptoms at the time of infection. Six months later, 28% still suffered from persistent joint pain, most commonly in the ankles. People older than 31 years were more likely to develop chronic pain.

These findings show that post-chikungunya chronic joint pain is common in Burkina Faso and highlight the need for follow-up care after the acute illness. Early identification of patients at risk could help prevent long-term disability and improve quality of life in affected communities.

## Introduction

Chikungunya is a viral disease transmitted to humans by *Aedes aegypti* and *Aedes albopictus* mosquitoes [1]. The chikungunya virus is an RNA virus of the genus *Alphavirus*, belonging to the *Togaviridae* family [1].

Since its first identification in 1955, numerous endemic foci have been documented in Europe, Asia, and Africa [2]. The seroprevalence of chikungunya in African countries is estimated at 7.28%, with Ghana showing the highest seroprevalence at 27.7%, followed by Sudan at 10.3% [3]. Conversely, Congo and Gabon report the lowest seroprevalences at 0.9% and 0.42%, respectively [3]. In 2023, Burkina Faso experienced its first chikungunya epidemic; Lim et al. reported that 29.1% of the general population of Ouagadougou, the capital of Burkina Faso, was seropositive for the virus [3].

The acute phase of chikungunya manifests as a classic triad combining sudden-onset fever, arthralgia or arthritis, and cutaneous eruption [4]. Articular manifestations represent the major complication of chikungunya, with approximately 90% of patients presenting with arthralgia or arthritis during the acute phase [4]. This acute phase may evolve toward disease resolution or toward a chronic phase marked by symptom persistence for weeks or even years [4]. These manifestations persist in 60% of patients and range from articular or peri-articular pain to polyarthritis that may mimic rheumatoid arthritis or spondyloarthritis [4].

Globally, arthralgia represents the principal osteoarticular manifestation of chikungunya, affecting 80 to 100% of patients [4]. In Réunion, 98% of patients presented with arthralgia, essentially polyarticular, affecting the wrists in 72% of cases and the ankles in 70.7% [5]. In Thailand, involvement was polyarticular in 92.7% of cases with ankle predominance [6]. However, to our knowledge, the osteoarticular manifestations of chikungunya remain poorly described in Africa, particularly in Burkina Faso.

Given the significant number of osteoarticular manifestations of chikungunya and the scarcity of African data on post-chikungunya chronic arthralgia, the objective of this study was to identify factors associated with post-chikungunya chronic arthralgia in Burkina Faso.

## Patients and methods

### Ethics statement

Anonymity and confidentiality of data were respected in accordance with the recommendations of the Declaration of Helsinki. Patients were enrolled after obtaining their informed written consent. The protocol was submitted for approval to the Ethics Committee for Health Research of Burkina Faso (CERS No. 2024-03-71).

### Study design

**Fig 1:**
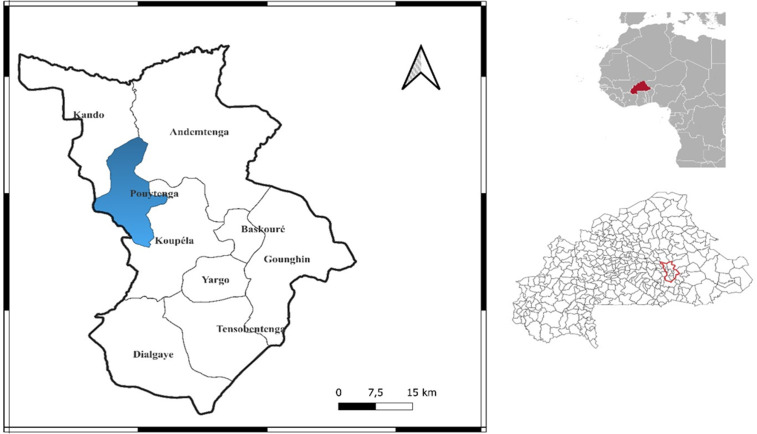
Geographic location of Pouytenga (study site), Burkina Faso. The map shows Pouytenga located approximately 140 kilometers east of Ouagadougou, the capital city. Map created by the authors using QGIS software (version 3.40.12) on August 18, 2025. Base map source: Natural Earth (public domain, http://www.naturalearthdata.com).

This was a descriptive and analytical cross-sectional study with ambispective data collection conducted from April 22 to December 28, 2024. The study was conducted at the medical center with surgical antenna of Pouytenga, located in the city of Pouytenga (Fig 1), 140 kilometers east of Ouagadougou, the capital city of Burkina Faso. This city was the main epidemic focus of chikungunya during the 2023 epidemic*. Pouytenga is a semi-urban district with an estimated population of approximately 150,000 inhabitants. Despite being a peripheral health facility,* the medical center with surgical antenna of Pouytenga *has established referral pathways to the national reference laboratory in Burkina Faso for molecular confirmation of suspected arboviral infections, enabling systematic RT-PCR testing of all suspected chikungunya cases during the epidemic period.*

### Population

The population consisted of patients seen in consultations or hospitalization at the Medical Center with Surgical Antenna of Pouytenga during the study period.

Patients aged 16 years and older who had chikungunya virus infection confirmed by biological results, notably RT-PCR, were included. Clinical records that were incomplete or those suffering from pre-existing chronic inflammatory rheumatism were excluded.

### Study variables

Data were collected from clinical records of patients seen in consultation or hospitalization. Telephone interviews and/or home visits were conducted to supplement information from incomplete clinical records. Post-chikungunya arthralgia was established when the patient answered “YES” at 6 weeks of disease evolution to the following question: “Do you have persistent joint pain or swelling related to chikungunya infection?” The search for post-chikungunya arthralgia after 6 months of evolution was performed exclusively by telephone interview after obtaining patient consent. The study variables were:

- Sociodemographic variables: age, gender, profession, marital status, and place of residence.
- Clinical variables: personal history, general signs (extra-articular signs), articular manifestations, duration of disease evolution, pain schedule (Mixed pain was defined as pain presenting both inflammatory features and mechanical features).
- Paraclinical variables: complete blood count, C-reactive protein, creatine phosphokinase, transaminases, RT-PCR; osteoarticular radiographs and ultrasounds. We used a molecular confirmation based on a specific and sensitive multiplex RT-PCR protocol designed for the rapid and simultaneous detection of Chikungunya virus [3]. Dengue co-infection was diagnosed using serological testing performed during the acute chikungunya episode, whereas malaria was identified through thick blood smear examination conducted at the same time. No diagnoses were based on patient self-report.
- Therapeutic variables: analgesics, non-steroidal anti-inflammatory drugs, corticosteroids, and other treatments.

### Statistical analysis

The collected data were analyzed using Epi Info 7.2.2.6 and Excel 2019 software. Quantitative variables were expressed as mean ± standard deviation. Qualitative variables were expressed as percentages and frequencies. In the bivariate analysis, Pearson’s Chi-square test or Fisher’s exact test was applied, as appropriate, to identify qualitative variables potentially associated with post-chikungunya chronic arthralgia. Variables with a p-value < 0.20 in bivariate analysis were selected for inclusion in the initial multivariate logistic regression model. This threshold was chosen to avoid excluding variables that could act as potential confounders. Before modeling, multicollinearity among independent variables was assessed using variance inflation factors (VIFs), and no significant collinearity was detected. The final logistic regression model was built using a stepwise backward elimination approach, retaining variables with p < 0.05 in the adjusted model. The goodness-of-fit of the final model was evaluated using the Hosmer–Lemeshow test, and the model performance was described by the Nagelkerke pseudo-R² value. Adjusted odds ratios with their 95% confidence intervals were calculated and presented for all variables included in the model, regardless of statistical significance.

## Results

### General population characteristics

**Fig 2:**
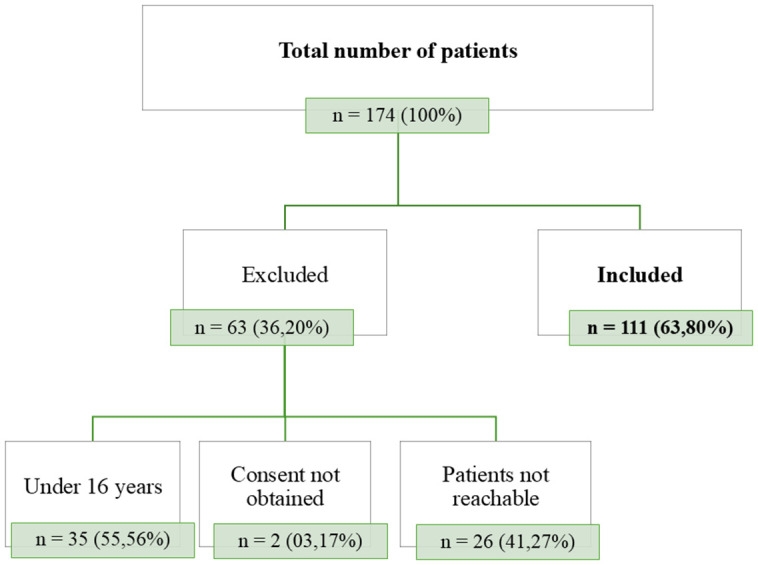
Study flow diagram. Detailed flow diagram illustrating the inclusion process of participants. The figure shows the total number of patients screened, the number excluded with reasons for exclusion, and the final number of patients retained.

We enrolled 174 patients. One hundred eleven patients with chikungunya were included in the study after exclusion of 63 (36.20%) patients. Among the excluded patients, 26 (41.27%) could not be reached and 2 (3.17%) did not give their consent. Thirty-five (55.56%) patients were under 16 years of age. Fig 2 shows the patient flow diagram. The mean age of patients was 31.18 years ± 11.27 years. Seventy-four (66.67%) patients were female; the sex ratio was 0.5. Forty (36.03%) patients were merchants and 39 (35.13%) patients were housewives. Twenty-eight (25.23%) patients had at least one comorbidity, including eleven (9.90%) with arterial hypertension and 4 (3.60%) with dengue co-infection. Table 1 groups the sociodemographic characteristics and comorbidities of our study population.

**Table 1:** Sociodemographic characteristics and comorbidities of the population.

Variable	Frequency (n)	Percentage
**Mean age ± SD (years)**	31.18 ± 11.27	
**Sex** Male	37	33.33
Female	74	66.66
**Occupation** Trader	40	36.04
Housewife	38	34.23
Student	16	14.41
Other profession	9	8.11
Civil servant	6	5.41
Retired	1	0.90
Farmer	1	0.90
**Comorbidities** Arterial hypertension	11	9.91
Malaria	6	5.41
Dengue	4	3.60
Hepatitis B	4	3.60
Diabetes mellitus	4	3.60
Peptic ulcer disease	4	3.60
HIV	3	2.70
Sickle cell disease	1	0.90
Asthma	1	0.90
**No comorbidity**	83	74.77

**Fig 3:**
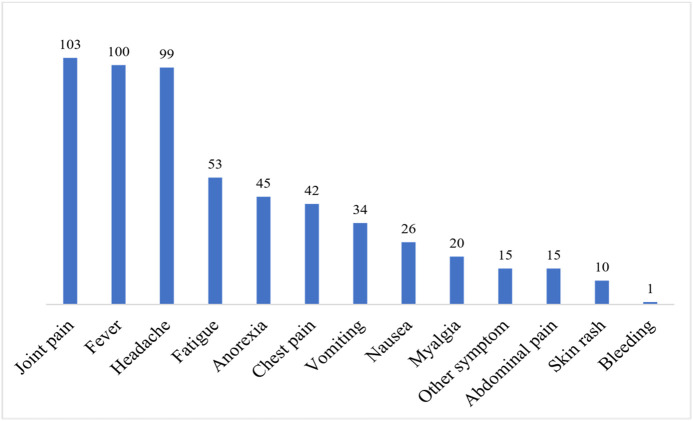
Vertical bar histogram of symptom distribution. Distribution of initial clinical manifestations reported by patients during the acute chikungunya episode. Each vertical bar represents the frequency of a specific symptom, expressed as the number of affected patients. This figure highlights the predominance of osteoarticular symptoms in the study population.

The mean duration of symptom evolution was 2.25 days ± 1.28 days. It refers to the average length of time during which patients continued to experience chikungunya related symptoms from onset until their resolution or until the 6 month evaluation point. At admission (M0), 103 (92.79%) patients had osteoarticular pain and 100 (90.09%) patients had fever (Fig 3).

Twenty-five (22.52%) patients underwent blood work including complete blood count and creatinine levels. All patients who underwent testing had normal creatinine levels. Twenty (80%) patients had normal leukocyte counts, 03 (12%) patients had leukopenia, and 02 (8%) patients had hyperleucocytosis. Other biological and imaging data were not documented.

Therapeutically, 107 (96.39%) patients received paracetamol treatment and 17 (15.31%) received intravenous rehydration. Antiemetics were prescribed to 9 patients (8.10%), and 2 patients (1.80%) received non-steroidal anti-inflammatory.

### Osteoarticular manifestations of chikungunya

One hundred three (92.79%) patients had arthralgia. The pain was classified as inflammatory in 92.23% of patients, defined by the presence of morning stiffness, improvement with movement, nocturnal or early-morning exacerbation, and/or clinically evident joint swelling. It was mixed in 6 (5.83%) patients and mechanical in 2 (1.94%) patients. The mean pain intensity using the visual analog scale (VAS) was 67.08 ± 9.96. Sixty-eight (66.02%) patients had morning stiffness exceeding 30 minutes. Thirty-five (31.53%) patients had spinal pain, including 29 (82.86%) with cervicalgia and 10 (28.57%) with lumbago. Eighty-two (79.61%) patients had arthralgia and 21 (20.39%) patients had arthritis. Involvement was symmetric in 76 (73.79%) patients, polyarticular in 68 (66.02%) patients, oligoarticular in 30 (29.13%) patients, and monoarticular in 5 (4.85%) patients. The ankle was affected in 78.37% of patients (n = 87) and the knee in 73.87% (n = 82). Table 2 represents the distribution of patients according to pain location.

**Table 2:** Distribution of patients according to pain location at M0.

Joint	Frequency (n)	Percentage
Ankle	87	84.46
Knee	82	79.61
Wrist	59	57.28
MTP	30	29.12
Elbow	29	28.15
Shoulder	29	28.15
Tarsus	25	24.27
Hip	24	23.30
MCP	24	23.32
PIP	20	19.41
DIP	11	10.67

*MCP: Metacarpophalangeal. PIP: Proximal Interphalangeal. DIP: Distal Interphalangeal. MTP: Metatarsophalangeal.*

### Evolution at 6 months and post-chikungunya chronic arthralgia

Of the 103 patients with initial joint involvement, 74 (71.84%) patients achieved complete remission after 6 months of evolution (M6).

**Fig 4:**
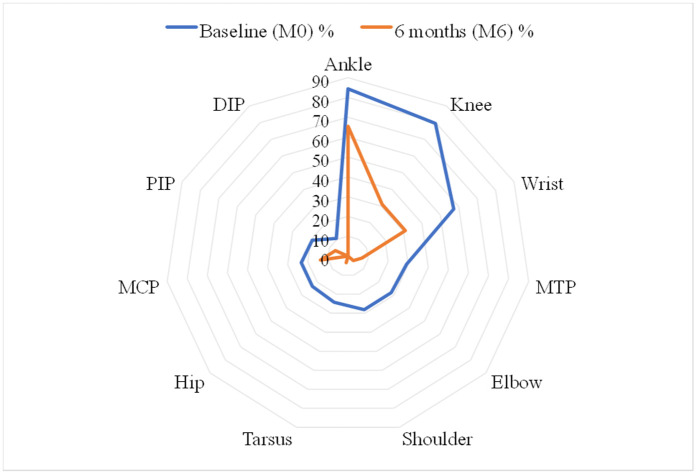
Radar chart of patient distribution according to pain location at M0 and M6 months of evolution. Each axis corresponds to a specific joint or joint group. *MCP: Metacarpophalangeal. PIP: Proximal Interphalangeal. DIP: Distal Interphalangeal. MTP: Metatarsophalangeal.*

Twenty-nine (28.16%) patients had post-chikungunya chronic arthralgia. There was no joint swelling. The pain was mechanical in 20 (68.97%) patients. It was mixed in 9 (31.03%) patients. The mean pain intensity using the visual analog scale (VAS) was 8.54 ± 4.44. Nineteen (65.52%) patients had persistent ankle pain (Table 3). Fig 4 represents the distribution of patients according to pain location at M0 and M6 of evolution.

**Table 3:** Distribution of patients according to pain location at 6 months of evolution.

Joint	Frequency (n)	Percentage
Ankle	19	65.52
Knee	9	31.03
Wrist	9	31.03
MCP	4	13.79
MTP	2	6.89
PIP	2	6.89
Tarsus	1	3.44
Elbow	1	3.44

*MCP: Metacarpophalangeal. PIP: Proximal Interphalangeal. DIP: Distal Interphalangeal. MTP: Metatarsophalangeal.*

### Factors associated with Post-chikungunya chronic arthralgia

In the bivariate analysis, age greater than 31 years was significantly associated with post-chikungunya chronic arthralgia (p < 0.001; OR = 4.75; 95% CI [1.90–11.87]). Among comorbidities, arterial hypertension was also associated with chronicity (p = 0.040; OR = 3.60; 95% CI [1.00–12.91]). Regarding clinical presentation, a polyarticular form tended to increase the risk of chronic arthralgia (p = 0.074; OR = 2.20; 95% CI [0.92–5.27]), while dengue co-infection showed a borderline association (p = 0.067; OR = 8.42; 95% CI [0.84–84.61]) (Table 4).

**Table 4:** Results of bivariate analysis of factors associated with Post-Chikungunya Chronic Arthralgia (Pchik-CA).

Variable	Pchik-CA Yes n (%)	Pchik-CA No n (%)	OR	CI à 95%	p-value
**Female sex**	18 (25.71%)	52 (74.29%)	0.69	0.28 – 1.70	0.422
**Age > 31 years**	17 (50.00%)	17 (50.00%)	4.75	1.90 – 11.87	**0.001**
**HIV infection**	1 (33.33%)	2 (66.67%)	1.29	0.11 – 14.75	1.000
**Hepatitis B infection**	1 (25.00%)	3 (75.00%)	0.85	0.08 – 8.47	1.000
**Dengue co-infection**	3 (75.00%)	1 (25.00%)	8.42	0.84 – 84.61	**0.067**
**Malaria infection**	1 (16.67%)	5 (83.33%)	0.49	0.06 – 4.41	1.000
**Arterial** **hypertension**	6 (54.55%)	5 (45.45%)	3.60	1.00 – 12.91	**0.040**
**Diabetes** **mellitus**	2 (50.00%)	2 (50.00%)	2.67	0.36 – 19.89	0.315
**Polyarticular** **form**	24 (35.29%)	44 (64.71%)	2.20	0.92 – 5.27	**0.074**
**Joint swelling**	8 (38.10%)	13 (61.90%)	1.79	0.65 – 4.91	0.256

All variables with a p-value < 0.20 in the bivariate analysis were included in the multivariate logistic regression model. Multicollinearity was assessed using variance inflation factors (VIFs), all below 1.13, indicating no significant collinearity among predictors.

In the multivariate analysis, only age greater than 31 years (adjusted OR = 6.22; 95% CI [2.35–16.47]; p < 0.001) and dengue co-infection (adjusted OR = 18.67; 95% CI [1.74–199.70]; p = 0.016) remained independently associated with post-chikungunya chronic arthralgia (Table 5). However, this finding must be interpreted with extreme caution given the very small number of dengue co-infected patients (n = 4) and the exceptionally wide confidence interval spanning two orders of magnitude, which indicates very low precision in this estimate. Arterial hypertension and polyarticular form were not retained in the final model after backward stepwise selection.

**Table 5:** Multivariate logistic regression analysis.

Variable	Crude OR (95% CI)	p-value (bivariate)	Adjusted OR (95% CI)*	p-value (adjusted)
**Final model variables**				
**Age > 31 years**	4.75 (1.90–11.87)	0.001	6.22 (2.35–16.47)	**0.001**
**Dengue infection**	8.42 (0.84–84.61)	0.067	18.67 (1.74–199.70)	**0.016**‡
**Variables eliminated during backward selection**				
**Arterial hypertension**	3.60 (1.00–12.91)	0.040	2.02 (0.54–7.60)†	0.324†
**Polyarticular form**	2.20 (0.92–5.27)	0.074	1.97 (0.79–4.88)†	0.173†

* Adjusted for variables with p < 0.20 in bivariate analysis using a backward stepwise logistic regression model.

† Variables excluded from the final model due to lack of statistical significance (p > 0.05).

Nagelkerke pseudo-R²: 0.237, AIC: 109.91; Variance Inflation Factors: all < 1.13 (no multicollinearity detected). ‡ The dengue co-infection estimate is based on only 4 patients and has very low precision as indicated by the extremely wide confidence interval spanning two orders of magnitude. This finding should be considered hypothesis-generating and requires validation in larger studies.

The final model demonstrated acceptable fit, with a Nagelkerke pseudo-R² of 0.237, indicating that approximately 23.7% of the variance in chronic arthralgia outcomes was explained by the included variables. The Akaike Information Criterion (AIC) was 109.91. The Hosmer–Lemeshow test could not be reliably computed due to limited data distribution across deciles, likely related to the modest sample size.

## Discussion

Our study aimed to identify factors associated with post-chikungunya chronic arthralgia during the first epidemic in Burkina Faso. To our knowledge, this is the first study devoted to evaluating predictive factors for chronicity of post-chikungunya articular manifestations in Africa. In our series, the frequency of osteoarticular manifestations was 92.79%, with arthralgia representing 79.61% of patients. This result is similar to the majority of studies conducted worldwide, demonstrating the high frequency of joint involvement during chikungunya [4–6].

In our study, arthralgia persisted in 28.16% of patients after 6 months of evolution. Our data differ from international literature, which generally reports higher chronicity rates. Indeed, Sissoko et al. in 2008 reported pain persistence in 57% of patients from Mayotte after 15 months of follow-up [7]. In India, Rahim et al. in 2016 found 36.28% chronic arthralgia, while Bouquillard et al. in 2018 on Réunion Island reported 83.1% after 32 months of follow-up [6,8]. The low frequency of chronic arthralgia reported in our study could be explained by the follow-up duration of our evaluation at 6 months. Although clinically relevant, this duration is relatively short compared to studies reporting extended follow-ups of 15 to 32 months. This difference could underestimate the true frequency of chronic manifestations, given that some patients may develop persistent symptoms beyond this period or present late recurrences. Additionally, the diversity of chikungunya virus strains could play a role in the variation in frequency of chronic manifestations [1]. Different viral strains in various geographical regions could present variable chronicity potential, thus influencing the long-term evolution of infected patients [1].

In multivariate analysis, age greater than 31 years increased the risk of developing persistent articular manifestations by 6.22 times (OR = 6.22; 95% CI [2.35–16.47]; p < 0.001). The association between age greater than 31 years and chronicity is explained by immunosenescence phenomena that reduce the organism’s adaptive immune response and favor low-grade chronic inflammation, conducive to arthralgia persistence and decreased tissue repair capacity [9]. Dengue co-infection also increased the risk of arthralgia chronicity by 18.67 times (OR = 18.67; 95% CI [1.74–199.70]; p = 0.016). This co-infection could lead to potentiation of the initial inflammatory response, creating increased cytokine mobilization that further damages synovial tissues [10]. However, it should be noted that dengue may also constitute a confounding factor in this association, given that this virus is itself capable of inducing chronic joint involvement, making it difficult to specifically attribute persistent symptoms to chikungunya or dengue in this context [11]. Nevertheless, this result should be interpreted with caution, as it is based on a very small number of co-infected patients (n = 4), leading to a wide confidence interval and limited statistical precision. The 95% confidence interval (1.74-199.70) spans two orders of magnitude, reflecting extremely low precision. Such a wide interval indicates substantial uncertainty around the true effect size and makes it impossible to reliably quantify the magnitude of any potential association. This finding should therefore be considered hypothesis-generating rather than conclusive, and requires validation in larger, adequately powered studies specifically designed to investigate the role of dengue-chikungunya co-infection in disease chronicity.

The literature describes several predictive factors for persistent articular manifestations [4–7]. Advanced age constitutes a consistently found factor, with variable thresholds according to studies ranging from 35 to 45 years [4–7]. Female gender is also frequently associated with chronicity, as is the severity of initial joint pain [6,12]. Other predictive factors for chronicity have been found in the literature, such as the presence of pre-existing arthritis and serology with high anti-IgG antibody titers [4–7]. Unlike many studies, we did not find significant associations with these other factors, possibly due to the limited size of our sample.

From a pathophysiological standpoint, the persistence of post-chikungunya articular manifestations involves several mechanisms. The presence of virus in joint tissues constitutes one of the main hypotheses. Studies have demonstrated the presence of viral RNA in synovial fluid and synovial biopsies from patients with chronic arthritis, suggesting persistent local viral replication that would maintain joint inflammation [13]. Autoimmune mechanisms represent another major pathophysiological pathway. Indeed, initial infection by chikungunya virus could trigger an autoimmune reaction through molecular mimicry, where antibodies directed against viral proteins would crossrecognize self-antigens, particularly at the level of synovial tissues [13,14]. This hypothesis is reinforced by symptom persistence even in the absence of viral detection. Finally, low-grade chronic inflammation also constitutes a suggested mechanism with prolonged activation of synovial macrophages and excessive production of proinflammatory cytokines such as IL-1β, TNF-α, IL-6 responsible for tissue lesions [15]. The pathophysiological mechanisms underlying persistent post-chikungunya articular manifestations may also involve NETosis, a process whereby neutrophils release extracellular traps containing neutrophil elastase and myeloperoxidase [16]. This mechanism, described in post-COVID-19 syndrome, could explain the chronicity of articular inflammation through several pathways. It’s the release of cryptic selfantigens serving as substrates for immunization, the induction of tissue damage with externalization of tissue-specific auto-antigens, and the release of damage-associated molecular patterns (DAMPs) perpetuating inflammation [16]. Limited efficacy of efferocytosis in clearing these neutrophil extracellular traps could maintain a low-grade inflammatory state conducive to arthralgia persistence [16]. This hypothesis is reinforced by the significant association observed between dengue co-infection and chronicity in our study, suggesting that more intense initial neutrophil hyperactivation could predispose to prolonged NETosis phenomena and the emergence of autoimmune processes. [16]

This study presents several strengths. It is the first study dedicated to post-chikungunya chronic arthralgia in Burkina Faso and in a sub-Saharan African context. Also, systematic biological confirmation by PCR of all included cases guarantees the reliability of chikungunya diagnosis. Our results allow identification of a patient profile at high risk of developing post-chikungunya chronic arthralgia. Patients older than 31 years, particularly in cases of dengue co-infection, require surveillance and prolonged follow-up beyond the acute phase. This early identification of risk factors could enable the implementation of preventive therapeutic strategies. Furthermore, the identification of dengue co-infection as a major risk factor underlines the importance of differential diagnosis and systematic search for co-infections in endemic areas of these two arboviruses.

However, several limitations must be considered in interpreting our results. First, insufficient data in medical records constitutes a major limitation. Indeed, virtually all patients lacked information on important paraclinical data. This absence of data limits the analysis of prognostic factors and complete characterization of the evolutionary profile. A major limitation of our study is the very small number of patients with dengue co-infection (n = 4), which resulted in an extremely wide confidence interval for this variable in the multivariate analysis. This wide interval, spanning two orders of magnitude, indicates very low precision and prevents any reliable quantification of the association between dengue co-infection and chronic arthralgia. While the observed association reached statistical significance, the small sample size and imprecise estimate mean this finding must be considered exploratory and requires confirmation in larger studies. Second, data on reassessment at 6 months were collected by self-declaration, which prevented verification of symptoms through objective clinical examination. In the case of subjective self-declaration, one cannot exclude memory bias leading to underestimation or overestimation of current or past symptoms. Finally, the loss-tofollow-up rate was high, reducing the statistical power of the study and potentially introducing selection bias.

## Conclusion

This study on post-chikungunya chronic arthralgia reveals that 28.16% of patients develop persistent arthralgia six months after acute infection. These are dominated by symmetric polyarthralgia primarily affecting the ankles. Age above 31 years was clearly identified as an independent risk factor for chronic post-chikungunya arthralgia, while the potential role of dengue co-infection requires further investigation in larger studies due to the very small sample size and low precision in our analysis. These identified factors constitute accessible surveillance tools to guide clinicians in early identification of patients at risk of chronicity. Beyond this 6-month follow-up, our cohort continues to be monitored to identify patients who may develop authentic chronic inflammatory rheumatism. Multicenter studies with extended follow-ups and larger sample sizes would be necessary to investigate progression and deepen understanding of chronicity mechanisms in the African context.
